# Preclinical evaluation of two ^68^Ga-siderophores as potential radiopharmaceuticals for *Aspergillus fumigatus* infection imaging

**DOI:** 10.1007/s00259-012-2110-3

**Published:** 2012-04-24

**Authors:** Milos Petrik, Gerben M. Franssen, Hubertus Haas, Peter Laverman, Caroline Hörtnagl, Markus Schrettl, Anna Helbok, Cornelia Lass-Flörl, Clemens Decristoforo

**Affiliations:** 1 Clinical Department of Nuclear Medicine, Innsbruck Medical University, Anichstrasse 35, 6020 Innsbruck, Austria; 2 Department of Nuclear Medicine, Radboud University Nijmegen Medical Center, Nijmegen, Netherlands; 3 Division of Molecular Biology/Biocenter, Innsbruck Medical University, Innsbruck, Austria; 4 Department of Hygiene, Microbiology and Social Medicine, Innsbruck Medical University, Innsbruck, Austria; 5 Laboratory of Experimental Medicine, Institute of Molecular and Translational Medicine, Palacky University, Olomouc, Czech Republic

**Keywords:** ^68^Ga-siderophores, *Aspergillus fumigatus*, Invasive aspergillosis, Infection imaging

## Abstract

**Purpose:**

Invasive pulmonary aspergillosis is mainly caused by *Aspergillus fumigatus*, and is one of the major causes of morbidity and mortality in immunocompromised patients. The mortality associated with invasive pulmonary aspergillosis remains high, mainly due to the difficulties and limitations in diagnosis. We have shown that siderophores can be labelled with ^68^Ga and can be used for PET imaging of *A. fumigatus* infection in rats. Here we report on the further evaluation of the most promising ^68^Ga-siderophore candidates, triacetylfusarinine (TAFC) and ferrioxamine E (FOXE).

**Methods:**

Siderophores were labelled with ^68^Ga using acetate buffer. Log *P*, protein binding and stability values were determined. Uptake by *A. fumigatus* was studied in vitro in cultures with high and low iron loads. In vivo biodistribution was determined in normal mice and an infection model was established using neutropenic rats inoculated with *A. fumigatus*. Static and dynamic μPET imaging was performed and correlated with CT images, and lung infection was evaluated ex vivo.

**Results:**

^68^Ga-siderophores were labelled with high radiochemical purity and specific activity. ^68^Ga-TAFC and ^68^Ga-FOXE showed high uptake by *A. fumigatus* in iron-deficient cultures. In normal mice, ^68^Ga-TAFC and ^68^Ga-FOXE showed rapid renal excretion with high metabolic stability. In the rat infection model focal lung uptake was detected by μPET with both compounds and increased with severity of the infection, correlating with abnormal CT images.

**Conclusion:**

^68^Ga-TAFC and ^68^Ga-FOXE displayed excellent in vitro stability and high uptake by *A. fumigatus*. Both compounds showed excellent pharmacokinetics, highly selective accumulation in infected lung tissue and good correlation with severity of disease in a rat infection model, which makes them promising agents for *A. fumigatus* infection imaging.

**Electronic supplementary material:**

The online version of this article (doi:10.1007/s00259-012-2110-3) contains supplementary material, which is available to authorized users.

## Introduction

Invasive fungal diseases are among the leading causes of morbidity and mortality in haematopoietic stem cell and solid organ transplant recipients, as well as in patients with solid tumours and haematological malignancies [1–3]. In recent years, an increase (from 19 % to 25 %) in the incidence of infections caused by opportunistic mould pathogens including *Aspergillus*, *Candida*, zygomycete, *Fusarium*, *Scedosporium* and *Acremonium* species has been observed [1, 3, 4], with invasive aspergillosis (IA) being the predominant infection [5, 6]. The mortality rate associated with IA, which is mainly caused by *Aspergillus fumigatus* and primarily affects the lungs, remains unacceptably high (30–95 %) [2, 3, 7]. Singh et al. have reported that an estimated 9.3–16.9 % of all deaths in transplant recipients in the first year can be attributable to IA [8, 9].

Early and accurate diagnosis of IA is critical for a favourable outcome, but is difficult to achieve with currently available methods [10, 11]. Current methods for the diagnosis of IA include prognostic factors, clinical signs, radiology and laboratory tests (e.g. galactomannan antigen, PCR, microscopy and culture) [10, 11]. However, most of these techniques lack sufficient specificity and/or sensitivity for early detection of IA. Identification of patients at high risk, appropriate prophylaxis, diagnostic surveillance, and early diagnosis remain important for improved patient management [11], and underline the need for specific and sensitive imaging methods for IA.

Iron is an essential nutrient and is also a key factor in the virulence of pathogenic microorganisms [12, 13]. In response to low iron availability, iron-dependent microorganisms have evolved different strategies to obtain iron. These strategies include the biosynthesis of low molecular mass iron chelators, termed siderophores, with extremely high affinity for ferric ions, which are employed for iron delivery by almost all bacteria and fungi (including *A. fumigatus*) as well as in some plants [14]. The major siderophore produced by *A. fumigatus* for iron acquisition is triacetylfusarinine C (TAFC). The importance of TAFC for *A. fumigatus* during virulence is reflected by the transcriptional upregulation of its biosynthesis and uptake during infection as well as the attenuation of virulence by inactivation of TAFC biosynthesis in murine IA models [15, 16]. *Aspergillus* recovers iron from iron-siderophore complexes via specific uptake mechanisms involving highly efficient siderophore transporters [17]. Remarkably, numerous fungi including *Aspergillus* species possess specific uptake systems not only for native siderophores, but also for siderophores synthesized exclusively by other fungi [18].

^68^Ga is a positron emitter that has recently become the subject of great interest for molecular imaging applications using PET [19]. It is readily available from a ^68^Ge/^68^Ga generator and has a suitably short half-life of 68 min. In addition, Ga^3+^ has comparable complex chemistry to Fe^3+^, and binds with high affinity to siderophores [20].

In a proof of principle study, we recently showed that a ^68^Ga-labelled TAFC can detect *A. fumigatus* infection in a rat animal model using PET imaging [20]. In a subsequent study, we characterized the in vitro and in vivo behaviour of selected siderophores [21], showing that besides ^68^Ga-TAFC, ^68^Ga-ferrioxamine E (^68^Ga-FOXE) also shows high uptake by *A. fumigatus* in culture and remains stable in vivo. In this study we compared these most promising candidates including investigations in a rat *A. fumigatus* infection model and μPET imaging.

## Materials and methods

### Chemicals

All commercially available reagents were of analytical grade and used without further purification. Siderophores were obtained from Genaxxon Bioscience (Ulm, Germany). ^68^Ga was eluted from a ^68^Ge/^68^Ga generator (IGG; Eckert & Ziegler, Berlin, Germany).

### Radiolabelling and in vitro studies

TAFC and FOXE were labelled with ^68^Ga using acetate buffer at room temperature for 15 min (TAFC) and at 80 °C for 20 min (FOXE). For all in vitro and in vivo studies, the pH of the final product was adjusted with 1.1 M sodium acetate to pH 6–7. The radiochemical purity, log *P*, protein binding and stability of ^68^Ga-siderophores in various media were determined, as described previously [20].

### Preparation of *A. fumigatus* cultures for in vitro uptake studies

The *Aspergillus* strain used for in vitro studies was *A. fumigatus* wild-type ATCC46645 (American Type Culture Collection) cultured at 37 °C in *Aspergillus* minimal medium, containing 1 % glucose as the carbon source, 20 mM glutamine as the nitrogen source, salts and trace elements, as described previously [22]. Iron-sufficient media contained 30 mM FeSO_4_. For preparation of iron-deficient media, iron addition was omitted. Iron-deficient conditions were verified by detection of extracellular siderophore production, which is suppressed by iron.

### In vitro uptake of ^68^Ga-siderophores by *A. fumigatus*

Uptake by *A. fumigatus* in iron-deficient and iron-sufficient cultures was studied. For the monitoring of uptake over time, ^68^Ga-siderophores (5 ng) were incubated in microbial media for 10, 20, 30, 45, 60 and 90 min at room temperature in 96-well plates (Millipore, Billerica, MA). For the monitoring of uptake blocking, excess of ferri-siderophore (Fe-TAFC or Fe-FOXE) and/or sodium azide was used. ^68^Ga-siderophores were incubated in iron-deficient and iron-sufficient media for 45 min at room temperature in 96-well plates. The incubation was interrupted in both cases by filtration of the medium and rapid rinsing with ice-cold TRIS buffer. The filters were collected and counted in a γ-counter.

### Preparation of *A. fumigatus* inoculum for rat infection model

The *A. fumigatus* (A29) isolate was grown on Sabouraud dextrose agar (BD) for 5 days at 37 °C, and the conidia were harvested in 2 ml of sterile NaCl by gently rubbing with a pipette tip. The conidia suspension was transferred into a sterile 50 ml plastic tube. After homogenization (vortex) and filtration (40 μm nylon cell strainer; BD), the suspension was counted in a Neubauer chamber and adjusted to the volitional concentration in the range 1 × 10^5^ to 1 × 10^9^ conidia per millilitre.

### Animal experiments

All animal experiments were conducted in accordance with regulations and guidelines of the Austrian and Dutch animal Protection laws and with the approval of the Austrian Ministry of Science (66011/42-II/10b/2009), and the institutional Animal Welfare Committee of the Radboud University Medical Centre Nijmegen (revised Dutch Act on Animal Experimentation, 1997). Animal studies were performed using Balb/c mice and Lewis rats (both Charles River Laboratories, Wilmington, MA).

### Biodistribution in normal mice

Normal noninfected Balb/c mice (female, 6 weeks old) were injected with ^68^Ga-siderophore (2 MBq and 0.1–0.2 μg of siderophore per mouse) into the tail vein. Animals were killed by cervical dislocation 30 min and 90 min after injection. The organs and tissues (blood, spleen, pancreas, stomach, intestines, kidneys, liver, heart, lungs, muscle and femur) were removed and radioactivity was counted in a γ-counter. The results are expressed as percentage of injected dose per gram of tissue.

### Metabolic stability

Urine, blood, liver and kidneys of normal Balb/c mice injected with ^68^Ga-siderophores and treated as described previously were collected 30 min after injection. The urine sample was directly injected onto the RP-HPLC column. Blood samples were precipitated with acetonitrile and centrifuged for 2 min, and the supernatant was injected onto the RP-HPLC column. Liver and kidneys were washed in the ice-cold TRIS buffer and liquidized using a mixer in a falcon tube containing 1 ml of TRIS buffer. The liver and kidney homogenates obtained were mixed with acetonitrile and centrifuged for 2 min, and the supernatant was injected onto the RP-HPLC column. In all cases 1 min fractions of the column eluate were collected and measured in a γ-counter. Samples were not collected at 90 min after injection because the measured activity was already low in the samples obtained 30 min after injection. All metabolic studies were performed using a previously described HPLC method [20].

### Rat infection models

#### Standard

Female or male Lewis rats (2–3 months old), weighing 200–250 g, were treated as described previously [20]. Briefly, the rats received repeated intraperitoneal injections of cyclophosphamide to induce neutropenia before *A. fumigatus* administration. To prevent bacterial superinfection, the animals were given antibiotics throughout the experiment. Fungal infection was established by intratracheal administration of 100–300 μl of *A. fumigatus* inoculum in various concentrations (1 × 10^5^ to 1 × 10^9^ conidia per millilitre). Animals were injected intravenously 1–3 days (depending on the severity of infection) after *A. fumigatus* administration with ^68^Ga-labelled siderophore (10–20 MBq and 1–2 μg of siderophore per rat). The rats were imaged or killed by overdosing with thiopental (Sandoz, Kundl, Austria) 2 h after injection. Various organs and tissues (blood, spleen, kidneys, liver and lungs) were removed and radioactivity was measured in a γ-counter. The excised organs were investigated for the presence of fungi, as described below.

#### Iron preload

Rats were treated as in the ‘standard’ infection model above, except that iron solution (FerMed; Medice, Iserlohn, Germany) 10 mg/kg was injected intraperitoneally three times (1 week, 4 days and 1 day) before *A. fumigatus* administration.

### ^68^Ga-siderophore imaging in the rat infection model

PET images were acquired with an Inveon animal PET/CT scanner (Siemens Preclinical Solutions, Knoxville, TN) with an intrinsic spatial resolution of 1.5 mm [23]. The animals were placed in a prone position. Static PET images were acquired over 30 min starting 30 min after intravenous injection of ^68^Ga-siderophore. Dynamic PET imaging was started upon injection and continued up to 60 min after injection. In addition, combined PET/CT scans were performed for anatomical reference. PET emission scans were acquired for 30 min, preceded by CT scans (spatial resolution 113 μm, 80 kV, 500 μA, exposure time 300 ms). After imaging, animals were killed by CO_2_/O_2_. Scans were reconstructed using Inveon Acquisition Workplace software (version 1.5; Siemens Preclinical Solutions, Knoxville, TN) using a 3-D ordered subset expectation maximization/maximum a posteriori (OSEM3D/MAP) algorithm with the following parameters: matrix 256 × 256 × 159, pixel size 0.43 × 0.43 × 0.8 mm^3^ and a MAP prior β-value of 1.5.

### In vitro cultures of excised organs

The excised organs were homogenized in a petri dish using a sterile surgical blade and transferred to Sabouraud dextrose agar (BD) plates. The plates were incubated at 37 °C and examined daily for 7 days. Colony-forming counts were recorded from all plates that showed growth. Severe infection was defined as the presence of severe fungal growth 1 day after incubation, mild infection was defined as the presence of minor growth up to 3 days after incubation, and no infection was defined as lack of growth within 1 week of incubation.

### Statistical analysis

Student’s *t*-test (level of significance, *P* < 0.05) was used to determine the significance of differences in the ex vivo and in vivo data. Analysis was performed using Microsoft Office Excel 2007.

## Results

### Radiolabelling and in vitro studies

**Fig. 1:**
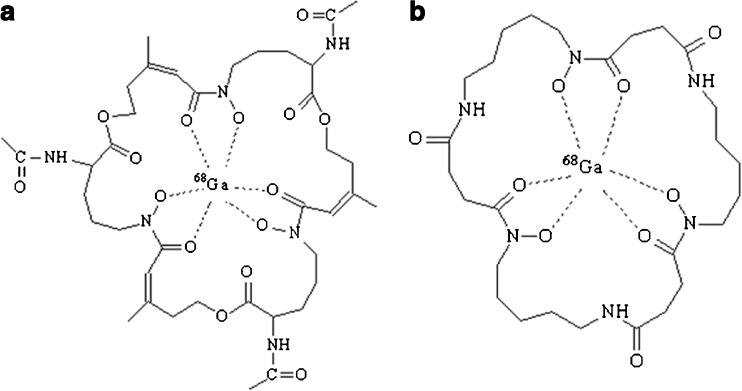
Chemical structures of ^68^Ga-TAFC (**a**) and ^68^Ga-FOXE (**b**)

^68^Ga-TAFC and ^68^Ga-FOXE (Fig. 1) were both labelled with high radiochemical purity (≥95 %). High specific activity labelling was achieved up to 9.2 × 10^4^ GBq/mmol for ^68^Ga-TAFC and 3.4 × 10^3^ GBq/mmol for ^68^ Ga-FOXE. Both compounds showed hydrophilic properties (log *P*_TAFC_ = −2.59, log *P*_FOXE_ = −1.65) with low values of protein binding even 120 min after incubation in human serum (1.21 % for ^68^Ga-TAFC, 0.53 % for ^68^Ga-FOXE). The stability of the studied compounds was tested in various media – human serum, 0.1 M FeCl_3_ and 6 mM DTPA. ^68^Ga-FOXE showed excellent stability (≥90 %) in all of the tested media. ^68^Ga-TAFC displayed comparable results, with the lowest stability (≥80 %) in 6 mM DTPA 120 min after incubation. A summary of the in vitro characteristics of both ^68^Ga-siderophores is given in Online Resource 1.

### In vitro uptake of ^68^Ga-siderophores by *A. fumigatus*

**Fig. 2:**
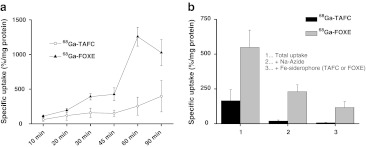
Specific uptake (difference between the uptake in iron-deficient and iron-sufficient cultures) of ^68^Ga-TAFC and ^68^Ga-FOXE by *A. fumigatus* over time (**a**) and 45 min after incubation blocked with excess of ferri-siderophore and/or sodium azide (**b**)

Uptake of ^68^Ga-TAFC and ^68^Ga-FOXE was highly dependent on the mycelial iron load. Both compounds showed rapid uptake by *A. fumigatus* in iron-deficient cultures, which could be blocked with excess of ferri-siderophore and/or sodium azide and was significantly lower (*P* < 0.05) in iron-sufficient media. Figure 2 shows ^68^Ga-TAFC and ^68^Ga-FOXE specific uptake over time (Fig. 2a) and specific uptake 45 min after incubation (Fig. 2b), which could be blocked with excess of ferri-siderophore and/or sodium azide in the *A. fumigatus* cultures.

### Biodistribution in normal Balb/c mice

In normal noninfected mice (Online Resource 2), ^68^Ga-TAFC showed rapid renal elimination and low blood levels as early as 30 min after injection (1.6 ± 0.37 %ID/g at 30 min). In a similar way, ^68^Ga-FOXE displayed fast renal elimination and relatively low blood levels 30 min after injection (2.4 ± 0.86 %ID/g). The main difference in biodistribution of the studied ^68^Ga-siderophores was in the significantly higher (*P* < 0.05) intestinal accumulation (see also liver values in Online Resource 2) of ^68^Ga-FOXE as compared to that of ^68^Ga-TAFC (4.6 ± 1.42 %ID/g at 30 min and 2.4 ± 0.85 %ID/g at 90 min for ^68^Ga-FOXE vs. 1.7 ± 1.04 %ID/g at 30 min and 1.0 ± 0.26 %ID/g at 90 min for ^68^Ga-TAFC).

### Metabolic stability

Both compounds showed high metabolic stability in urine, blood and kidney homogenate. In all cases almost only intact ^68^Ga-TAFC and ^68^Ga-FOXE were detected (the lowest values of administered ^68^Ga-TAFC and ^68^Ga-FOXE were 96.7 % and 95.9 % in kidney homogenate and urine, respectively). The major difference in the stability between the two ^68^Ga-siderophores was observed in liver homogenate. ^68^Ga-FOXE showed significantly higher (*P* < 0.05) metabolism than ^68^Ga-TAFC (^68^Ga-FOXE 71.3 % intact vs. ^68^Ga-TAFC 98.1 % intact on HPLC analysis). A summary of metabolic stabilities of the studied ^68^Ga-siderophores is given in Online Resource 3.

### Biodistribution of ^68^Ga-siderophores in rats – *A. fumigatus* infection model

**Fig. 3:**
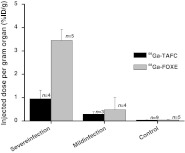
Ex vivo uptake in the lungs 2 h after injection of ^68^Ga-TAFC or ^68^Ga-FOXE measured in a γ-counter

In summary, 20 rats were treated with ^68^Ga-TAFC and 19 rats with ^68^Ga-FOXE. For ^68^Ga-TAFC, five rats developed severe and five rats mild lung infection, and ten rats showed no signs of infection. In the case of ^68^Ga-FOXE, six rats developed severe lung infection and six rats mild lung infection, and seven rats showed no sign of infection. In the severely infected animals, high levels of ^68^Ga-siderophores accumulated in the infected lungs, whereas in the noninfected animals, ^68^Ga-siderophores were rapidly excreted via the kidneys with low levels of accumulation in other organs. A significant difference (*P* < 0.05) between mildly infected and noninfected rats was observed. Figure 3 shows lung uptake values in the different groups of rats 2 h after administration of ^68^Ga-siderophores. The highest uptake was observed in severely infected rats injected with ^68^Ga-FOXE (3.45 ± 1.00 %ID/g, *n* = 5), followed by 0.95 ± 0.37 %ID/g (*n* = 4) in severely infected rats injected with ^68^Ga-TAFC. In the group of mildly infected animals, ^68^Ga-FOXE again showed slightly higher uptake 0.48 ± 0.54 %ID/g (*n* = 4) in comparison with ^68^Ga-TAFC (0.29 ± 0.12 %ID/g; *n* = 3). The control group showing no infection displayed similar results for both ^68^Ga-siderophores (0.04 ± 0.02 %ID/g for ^68^Ga-FOXE, *n* = 5; 0.04 ± 0.01 %ID/g for ^68^Ga-TAFC, *n* = 9). Rats pretreated with iron showed a comparable dependence of uptake on the severity of infection, with absolute values being somewhat lower than in the nonpretreated group, in particular for ^68^Ga-FOXE; however, none of the differences was statistically significant (*P* < 0.05). Online Resource 4 shows a comparison of lung uptake and target/non target ratios of ^68^Ga-siderophores in the different rat infection models.

### ^68^Ga-siderophore imaging in rats

**Fig. 4:**
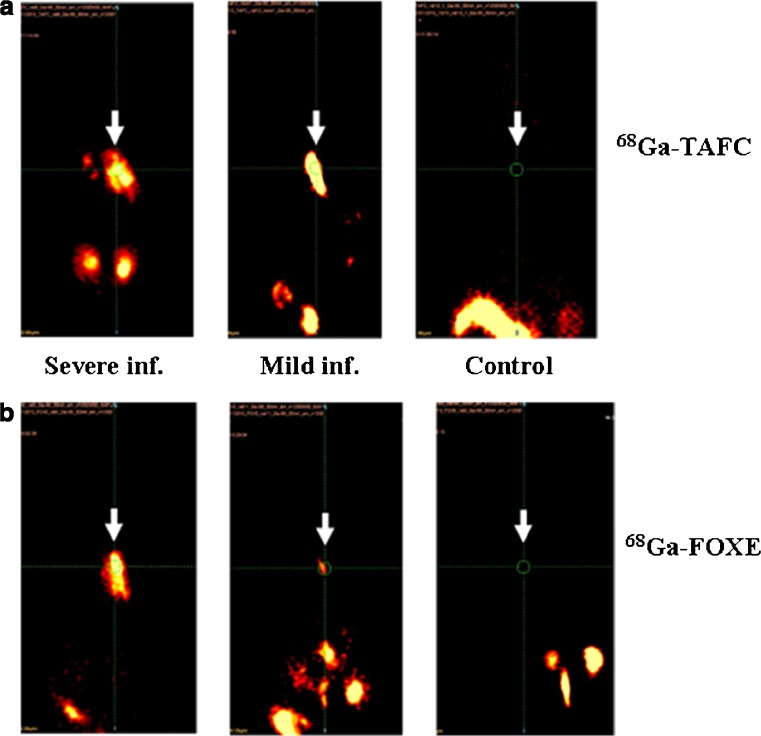
Static PET scans (30 min after injection) in severely infected rats (*left*), mildly infected rats (*centre*) and noninfected rats (*right*) show different levels of accumulation of ^68^Ga-TAFC (**a**) and ^68^Ga-FOXE (**b**) in infected lungs (*arrows*) depending on the severity of infection. No infection in non-infected animals is seen

**Fig. 5:**
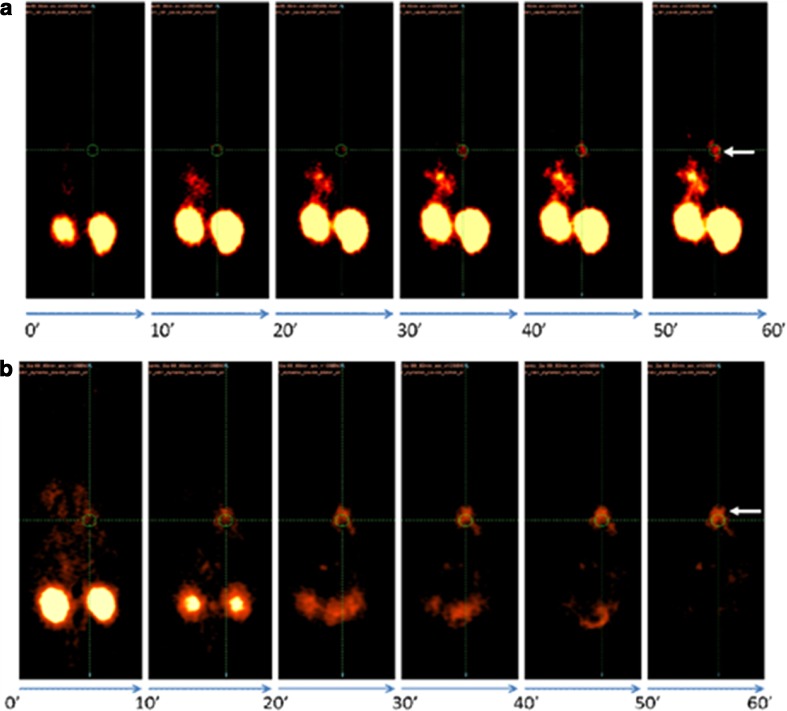
Dynamic PET images in infected rats show rapid uptake and no release of ^68^Ga-TAFC (**a**) and ^68^Ga-FOXE (**b**) in infected lung tissue (*arrows*) over time

**Fig. 6:**
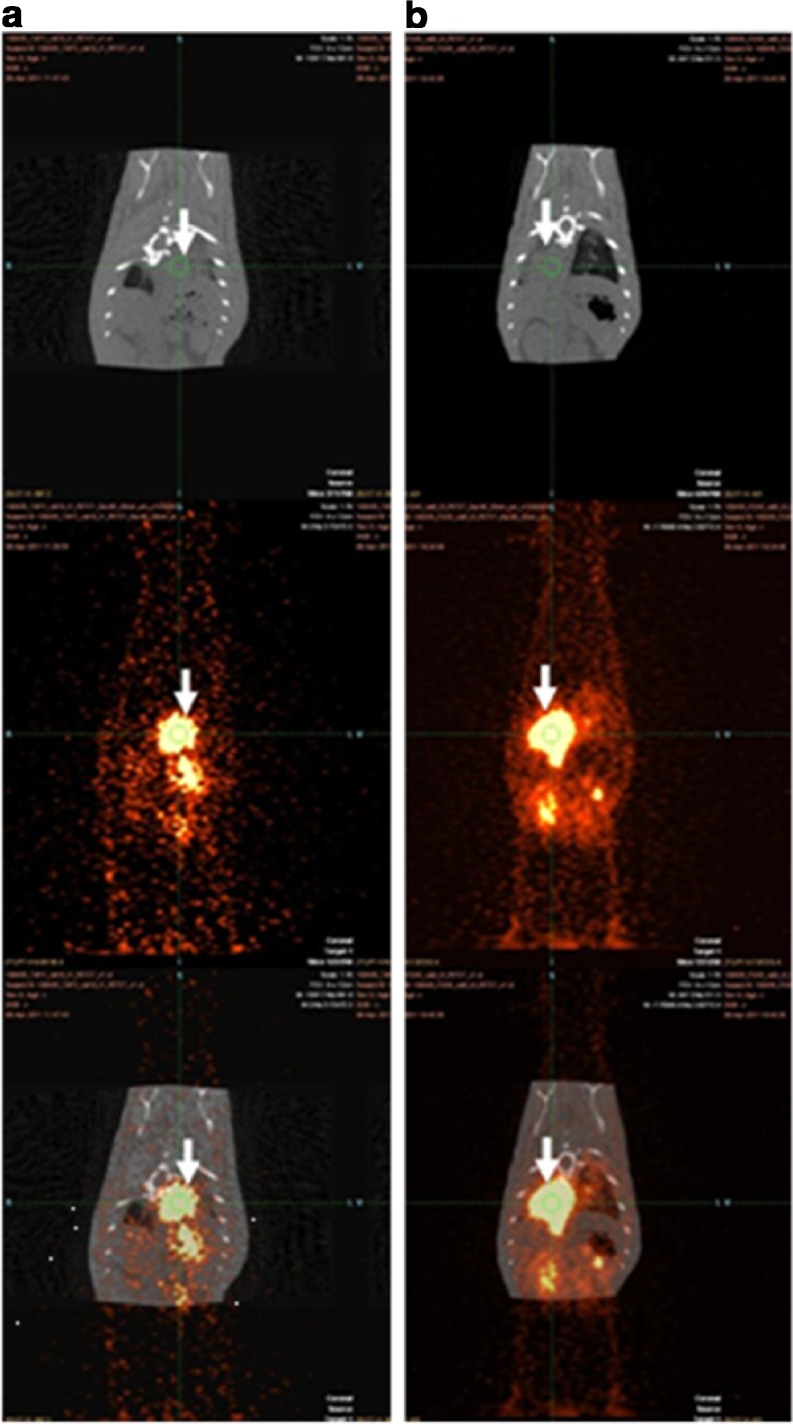
PET and CT scans correlating accumulation of ^68^Ga-TAFC (**a**) and ^68^Ga-FOXE (**b**) in rats infected with *A. fumigatus*(*top* CT scan, *centre* PET scan, *bottom* fused CT and PET image)

MicroPET imaging in the rat infection model showed rapid focal accumulation of ^68^Ga-siderophores in the lungs, which increased with the severity of infection (Fig. 4). No uptake in the lung region was detected in noninfected animals in which the only visible organs were the kidneys and bladder (Fig. 4). Dynamic imaging (see Fig. 5 for ^68^Ga-FOXE) in severely infected rats revealed uptake in the infected lung area as early as 10–20 min after injection with improved contrast over time without detectable washout over the whole imaging period (60 min), whereas the activity rapidly accumulated in kidneys and decreased over time. Figure 6 shows a correlation between PET and CT scans for both compounds under study. In CT scans of severely infected rats the changes in infected tissue were visible as grey areas in the lung region that fully corresponded with radioactivity lung accumulation in PET scans. Fusing images of both modalities revealed matching uptake, clearly visible in the fused images. Target/non-target ratios as well as SUV values in infected animals were calculated from all imaging studies performed (*n* = 13) and were 5.81 ± 6.05 and 0.78 ± 0.75 for ^68^Ga-TAFC and 6.64 ± 2.91 and 1.00 ± 0.81 for ^68^Ga-FOXE, respectively (see Online Resource 5), showing no significant difference (*P* < 0.05) between the two compounds in terms of quantitative uptake behaviour.

## Discussion

The need for novel approaches to the imaging of IA is reflected by the number of radiopharmaceuticals that have been described and proposed for this application [24, 25]. Radiopharmaceuticals for which clinical applications have been proposed include ^67^Ga-citrate [26], but it has known limitations in terms of pharmacokinetics, sensitivity and specificity, as it is a general marker for imaging malignancies and inflammatory processes. Even though ^18^F-FDG has also recently been proposed as an imaging agent for IA [27], it has comparable limitations, in particular related to the low specificity for imaging glucose metabolism. Various attempts have been made to develop more specific radiopharmaceuticals for this application, including ^99m^Tc-labelled polyethyleneglycol liposomes [28], ^99m^Tc-interleukin-8 [29], ^99m^Tc-fluconazole [30] and ^99m^Tc-antimicrobial peptides (e.g. ubiquicidin) [30, 31]. None of these agents has proven to show specific uptake mechanisms in *Aspergillus* species and none has entered clinical trials. Recently a hypha-binding peptide (c(CGGRLGPFC)-NH_2_) labelled with ^111^In has been described [32] potentially having higher specificity, but further evaluation towards clinical application has not been reported.

The use of a ^68^Ga-labelled siderophore that is actively taken up via specific iron transporters by the microorganism acquiring iron during the course of infection holds the potential of a unique and specific way to image IA. In a proof of principle study [20], we have shown that siderophores can be labelled with ^68^Ga with high affinity and stability in biological systems. In vitro energy-dependent uptake of ^68^Ga-siderophores in *A. fumigatus* was observed and preliminary in vivo studies were performed, proving the potential of ^68^Ga-labelled siderophores for infection imaging. After the promising study with ^68^Ga-TAFC in the rat infection model, we focused on the selection, characterization and optimization of the most promising candidates for diagnostic applications as a basis for clinical implementation of PET (PET/CT) in imaging of fungal infections [21].

In this study we compared two ^68^Ga-labelled siderophores as the most promising candidates from previous studies [20, 21] and evaluated their potential as radiopharmaceuticals for IA imaging. Both ^68^Ga-FOXE and ^68^Ga-TAFC showed hydrophilic properties, low protein binding and high in vitro stability. In vitro studies showed rapid and high uptake by *A. fumigatus* in iron-deficient media, which could be blocked with excess of ferri-siderophore or sodium azide. Both compounds showed excellent pharmacokinetic properties with high metabolic stability. Nevertheless, ^68^Ga-FOXE showed significantly lower metabolic stability in the liver than ^68^Ga-TAFC, which could explain the higher accumulation of radioactivity in the liver and intestinal tissue observed in biodistribution studies of Balb/c mice. This was confirmed in μPET imaging of rats. Both ^68^Ga-siderophores showed highly selective accumulation in infected lung which was shown to be correlated with severity of disease in the rat infection model using μPET or μPET/CT. Even though ^68^Ga-FOXE had higher uptake values in biodistribution studies indicating a potentially higher sensitivity, PET/CT imaging did not show significant differences either in uptake or in target/non-target ratios.

Today CT is the standard imaging technique for the detection of pulmonary infections [33, 34], and previous studies have shown that the halo sign is indicative of pulmonary aspergillosis in neutropenic patients [35]. However, CT scans may not allow differentiation between *Aspergillus* and other pathogenic fungi [36] and CT has limited specificity and predictive value, especially in non-neutropenic stem-cell transplant recipients [36, 37]. The combination with PET could provide additional functional information on the lesion detected, thereby increasing sensitivity and specificity of patient imaging within one diagnostic procedure. The effectiveness of combined PET and CT imaging is illustrated in Fig. 6. The PET images of both ^68^Ga-siderophores in rats infected with *A. fumigatus* show abnormal uptake of radiotracer in the thoracic area. The fused PET/CT image permits precise localization of the lung tissue affected by *A. fumigatus* infection. We found matching uptake in pathological areas on CT images with the accumulation of our investigated ^68^Ga-siderophores in the infected lung areas. The combination of PET and ^68^Ga-labelled siderophores (TAFC or FOXE) and CT therefore holds potential for early detection of invasive fungal infections.

In the clinical setting, one of the risk factors for IA in immunocompromised patients is a high iron load [38], which is frequently occurs due to blood transfusions or iron supplementation during the course of their underlying disease. As ^68^Ga-siderophores mimic iron-transporting mechanisms in microorganisms, we wanted to see whether an iron preload would have an effect on the uptake of ^68^Ga-labelled siderophores in the IA rat model. In the small series of animals tested, no significant decrease in uptake of either ^68^Ga-TAFC or ^68^Ga-FOXE in fungal infection could be observed indicating that iron supply does not influence uptake of the tracers. Another important factor in judging the suitability of this imaging approach is the selectivity of ^68^Ga-siderophores for fungal infections. We are currently investigating this issue in an ongoing study to determine the uptake of these ^68^Ga-siderophores by a variety of microorganisms, which will help in choosing the optimal candidate for noninvasive detection of fungal infections by PET in a clinical setting.

### Conclusion

Our study showed that both ^68^Ga-labelled TAFC and FOXE are very promising agents for detection of IA with high sensitivity. The high metabolic stability, favourable pharmacokinetics with rapid renal excretion and high specific uptake in *A. fumigatus* cultures were confirmed in imaging studies in a rat IA model that showed high focal uptake in infected lung tissue corresponding to pathological findings seen on CT. ^68^Ga-TAFC showed advantages in terms of radiolabelling and a somewhat higher metabolic stability, and ^68^Ga-FOXE showed a trend towards higher uptake in infected tissue. Only currently ongoing selectivity studies will enable selection of the optimal candidate for potential clinical applications.

## Electronic supplementary material

Below are the links to the electronic supplementary material.

Online Resource 1 In vitro characteristics of ^68^Ga-TAFC and ^68^Ga-FOXE (DOCX 15.8 kb)

Online Resource 2 Biodistribution of ^68^Ga-TAFC and ^68^Ga-FOXE in normal (noninfected) mice 30 and 90 min after injection (DOCX 57 kb)

Online Resource 3 Metabolic stability of ^68^Ga-TAFC and ^68^Ga-FOXE in normal Balb/c mice 30 min after injection. (DOCX 14 kb)

Online Resource 4 Comparison of lung uptake and ratios in organs of interest of ^68^Ga-siderophores in different groups of rat infection model (DOCX 17.0 kb)

Online Resource 5 Comparison of target/non-target (lung) ratios and SUVs of the studied ^68^Ga-siderophores in infected rats (DOCX 14 kb)
