# Supplementary material for: Preclinical evaluation of two 68Ga-siderophores as potential radiopharmaceuticals for Aspergillus fumigatus infection imaging
Source: Eur J Nucl Med Mol Imaging. 2012 Apr 24;39(7):1175–83. doi: 10.1007/s00259-012-2110-3 (PMC3369139; doi:10.1007/s00259-012-2110-3)
Supplement: Supplementary file 1 — In vitro characteristics of 68Ga-TAFC and 68Ga-FOXE (DOCX 15.8 kb) [file 259_2012_2110_MOESM1_ESM.docx]

| **^68^Ga-siderophore** | **Radiochemical purity (%)** | **Log *P*** | **Incubation time (min)** | **Protein binding (%)** | **Stability in human serum (%)** | **Stability in 0.1-M FeCl_3_ (%)** | **Stability in 6-mM DTPA (%)** |
| --- | --- | --- | --- | --- | --- | --- | --- |
| ^68^Ga-TAFC |  | −2.59 | 30 | 0.47 | 99.9 | 99.4 | 85.0 |
|  | ≥95 |  | 60 | 0.76 | 99.9 | 98.5 | 84.7 |
|  |  |  | 120 | 1.21 | 99.9 | 99.3 | 81.8 |
|  |  |  |  |  |  |  |  |
| ^68^Ga-FOXE |  | −1.65 | 30 | 0.27 | 99.9 | 92.9 | 94.3 |
|  | ≥95 |  | 60 | 0.24 | 99.9 | 91.8 | 93.8 |
|  |  |  | 120 | 0.53 | 99.9 | 94.5 | 93.2 |

**Online Resource 1** *In vitro* characteristics of ^68^Ga-TAFC and ^68^Ga-FOXE

Preclinical evaluation of two ^68^Ga-siderophores as potential radiopharmaceuticals for *Aspergillus fumigatus* infection imaging

European Journal of Nuclear Medicine and Molecular Imaging

Milos Petrik · Gerben M. Franssen · Hubertus Haas · Caroline Hörtnagl · Markus Schrettl · Anna Helbok · Cornelia Lass-Flörl · Peter Laverman · Clemens Decristoforo

Corresponding authors:

Milos Petrik

Clinical Department of Nuclear Medicine, Anichstrasse 35, A-6020 Innsbruck, Austria

Tel: +4351250480958; Fax: +435125046780951; Email: [milospetrik@seznam.cz](mailto:milospetrik@seznam.cz)

Clemens Decristoforo

Clinical Department of Nuclear Medicine, Anichstrasse 35, A-6020 Innsbruck, Austria

Tel: +4351250480951; Fax: +435125046780951; Email: [Clemens.Decristoforo@uki.at](mailto:Clemens.Decristoforo@uki.at)
