# Supplementary material for: Preclinical evaluation of two 68Ga-siderophores as potential radiopharmaceuticals for Aspergillus fumigatus infection imaging
Source: Eur J Nucl Med Mol Imaging. 2012 Apr 24;39(7):1175–83. doi: 10.1007/s00259-012-2110-3 (PMC3369139; doi:10.1007/s00259-012-2110-3)
Supplement: Supplementary file 2 — Biodistribution of 68Ga-TAFC and 68Ga-FOXE in normal (noninfected) mice 30 and 90 min after injection (DOCX 57 kb) [file 259_2012_2110_MOESM2_ESM.docx]

**Online Resource 2** Biodistribution of ^68^Ga-TAFC and ^68^Ga-FOXE in normal (non-infected) mice 30 and 90 min after injection

Preclinical evaluation of two ^68^Ga-siderophores as potential radiopharmaceuticals for *Aspergillus fumigatus* infection imaging

European Journal of Nuclear Medicine and Molecular Imaging

Milos Petrik · Gerben M. Franssen · Hubertus Haas · Caroline Hörtnagl · Markus Schrettl · Anna Helbok · Cornelia Lass-Flörl · Peter Laverman · Clemens Decristoforo

Corresponding authors:

Milos Petrik

Clinical Department of Nuclear Medicine, Anichstrasse 35, A-6020 Innsbruck, Austria

Tel: +4351250480958; Fax: +435125046780951; Email: [milospetrik@seznam.cz](mailto:milospetrik@seznam.cz)

Clemens Decristoforo

Clinical Department of Nuclear Medicine, Anichstrasse 35, A-6020 Innsbruck, Austria

Tel: +4351250480951; Fax: +435125046780951; Email: [Clemens.Decristoforo@uki.at](mailto:Clemens.Decristoforo@uki.at)
