# Supplementary material for: Preclinical evaluation of two 68Ga-siderophores as potential radiopharmaceuticals for Aspergillus fumigatus infection imaging
Source: Eur J Nucl Med Mol Imaging. 2012 Apr 24;39(7):1175–83. doi: 10.1007/s00259-012-2110-3 (PMC3369139; doi:10.1007/s00259-012-2110-3)
Supplement: Supplementary file 3 — Metabolic stability of 68Ga-TAFC and 68Ga-FOXE in normal Balb/c mice 30 min after injection. (DOCX 14 kb) [file 259_2012_2110_MOESM3_ESM.docx]

| **^68^Ga-siderophore** | **Stability in urine (%)** | **Stability in blood (%)** | **Stability in liver homogenate (%)** | **Stability in kidney homogenate (%)** |
| --- | --- | --- | --- | --- |
| ^68^Ga-TAFC | 97.6 | 98.7 | 98.1 | 96.7 |
| ^68^Ga-FOXE | 95.9 | 97.7 | 71.3 | 97.6 |

**Online Resource 3** Metabolic stability of ^68^Ga-TAFC and ^68^Ga-FOXE in normal Balb/c mice 30 min p.i.

Preclinical evaluation of two ^68^Ga-siderophores as potential radiopharmaceuticals for *Aspergillus fumigatus* infection imaging

European Journal of Nuclear Medicine and Molecular Imaging

Milos Petrik · Gerben M. Franssen · Hubertus Haas · Caroline Hörtnagl · Markus Schrettl · Anna Helbok · Cornelia Lass-Flörl · Peter Laverman · Clemens Decristoforo

Corresponding authors:

Milos Petrik

Clinical Department of Nuclear Medicine, Anichstrasse 35, A-6020 Innsbruck, Austria

Tel: +4351250480958; Fax: +435125046780951; Email: [milospetrik@seznam.cz](mailto:milospetrik@seznam.cz)

Clemens Decristoforo

Clinical Department of Nuclear Medicine, Anichstrasse 35, A-6020 Innsbruck, Austria

Tel: +4351250480951; Fax: +435125046780951; Email: [Clemens.Decristoforo@uki.at](mailto:Clemens.Decristoforo@uki.at)
