# Supplementary material for: Preclinical evaluation of two 68Ga-siderophores as potential radiopharmaceuticals for Aspergillus fumigatus infection imaging
Source: Eur J Nucl Med Mol Imaging. 2012 Apr 24;39(7):1175–83. doi: 10.1007/s00259-012-2110-3 (PMC3369139; doi:10.1007/s00259-012-2110-3)
Supplement: Supplementary file 4 — Comparison of lung uptake and ratios in organs of interest of 68Ga-siderophores in different groups of rat infection model (DOCX 17.0 kb) [file 259_2012_2110_MOESM4_ESM.docx]

| **Siderophore** | **Rat infection model** | **Severe infection (%ID/g - lungs) (mean ± sd)** | **Mild infection (%ID/g - lungs) (mean ± sd)** | **Control (%ID/g - lungs) (mean ± sd)** | **Ratios in organs of interest** | **Severe infection (mean ± sd)** | **Mild infection (mean ± sd)** | **Control (mean ± sd)** |
| --- | --- | --- | --- | --- | --- | --- | --- | --- |
| ^68^Ga-TAFC | Standard | 0.95 ± 0.37 (*n*=4) | 0.29 ± 0.12 (*n*=3) | 0.04 ± 0.01 (*n*=9) | Lung/blood | 15.11 ± 11.37 (*n*=4) | 3.82 ± 2.89 (*n*=3) | 1.34 ± 0.22 (*n*=9) |
|  |  |  |  |  | Lung/liver | 14.29 ± 6.10 (*n*=4) | 4.81 ± 2.70 (*n*=3) | 0.84 ± 0.48 (*n*=9) |
|  |  |  |  |  | Lung/kidneys | 1.40 ± 0.76 (*n*=4) | 0.16 ± 0.14 (*n*=3) | 0.07 ± 0.03 (*n*=9) |
|  | Iron preload | 0.64 (*n*=1) | 0.27 (*n*=2) | 0.04 (*n*=1) | Lung/blood | 1.41 (*n*=1) | 5.35 (*n*=2) | 0.99 (*n*=1) |
|  |  |  |  |  | Lung/liver | 3.49 (*n*=1) | 4.53 (*n*=2) | 0.76 (*n*=1) |
|  |  |  |  |  | Lung/kidneys | 0.07 (*n*=1) | 0.31 (*n*=2) | 0.08 (*n*=1) |
| ^68^Ga-FOXE | Standard | 3.45 ± 1.00 (*n*=5) | 0.48 ± 0.54 (*n*=4) | 0.04 ± 0.02 (*n*=5) | Lung/blood | 62.35 ± 77.98 (*n*=5) | 5.45 ± 5.57 (*n*=4) | 1.48 ± 0.33 (*n*=5) |
|  |  |  |  |  | Lung/liver | 47.79 ± 39.48 (*n*=5) | 5.45 ± 5.85 (*n*=4) | 0.87 ± 0.27 (*n*=5) |
|  |  |  |  |  | Lung/kidneys | 3.33 ± 2.85 (*n*=5) | 0.38 ± 0.42 (*n*=4) | 0.07 ± 0.02 (*n*=5) |
|  | Iron preload | 1.72 (*n*=1) | 0.14 (*n*=2) | 0.04 (*n*=2) | Lung/blood | 2.41 (*n*=1) | 0.81 (*n*=2) | 0.64 (*n*=2) |
|  |  |  |  |  | Lung/liver | 5.93 (*n*=1) | 1.17 (*n*=2) | 0.58 (*n*=2) |
|  |  |  |  |  | Lung/kidneys | 0.11 (*n*=1) | 0.12 (*n*=2) | 0.06 (*n*=2) |

**Online Resource 4** Comparison of lung uptake and ratios in organs of interest of ^68^Ga-siderophores in different groups of rat infection model

Preclinical evaluation of two ^68^Ga-siderophores as potential radiopharmaceuticals for *Aspergillus fumigatus* infection imaging

European Journal of Nuclear Medicine and Molecular Imaging

Milos Petrik · Gerben M. Franssen · Hubertus Haas · Caroline Hörtnagl · Markus Schrettl · Anna Helbok · Cornelia Lass-Flörl · Peter Laverman · Clemens Decristoforo

Corresponding authors:

Milos Petrik

Clinical Department of Nuclear Medicine, Anichstrasse 35, A-6020 Innsbruck, Austria

Tel: +4351250480958; Fax: +435125046780951; Email: [milospetrik@seznam.cz](mailto:milospetrik@seznam.cz)

Clemens Decristoforo

Clinical Department of Nuclear Medicine, Anichstrasse 35, A-6020 Innsbruck, Austria

Tel: +4351250480951; Fax: +435125046780951; Email: [Clemens.Decristoforo@uki.at](mailto:Clemens.Decristoforo@uki.at)
