# Supplementary material for: Preclinical evaluation of two 68Ga-siderophores as potential radiopharmaceuticals for Aspergillus fumigatus infection imaging
Source: Eur J Nucl Med Mol Imaging. 2012 Apr 24;39(7):1175–83. doi: 10.1007/s00259-012-2110-3 (PMC3369139; doi:10.1007/s00259-012-2110-3)
Supplement: Supplementary file 5 — Comparison of target/non-target (lung) ratios and SUVs of the studied 68Ga-siderophores in infected rats (DOCX 14 kb) [file 259_2012_2110_MOESM5_ESM.docx]

| **Siderophore** | **Target/non-target ratio** | **SUV** |
| --- | --- | --- |
| ^68^Ga-TAFC | 5.81 ± 6.05 (*n*=13) | 0.78 ± 0.75 (*n*=10) |
| ^68^Ga-FOXE | 6.64 ± 2.91 (*n*=13) | 1.00 ± 0.81 (*n*=10) |

**Online Resource 5** Comparison of target/non-target (lungs) ratios and SUVs of studied ^68^Ga-siderophores in infected rats

Preclinical evaluation of two ^68^Ga-siderophores as potential radiopharmaceuticals for *Aspergillus fumigatus* infection imaging

European Journal of Nuclear Medicine and Molecular Imaging

Milos Petrik · Gerben M. Franssen · Hubertus Haas · Caroline Hörtnagl · Markus Schrettl · Anna Helbok · Cornelia Lass-Flörl · Peter Laverman · Clemens Decristoforo

Corresponding authors:

Milos Petrik

Clinical Department of Nuclear Medicine, Anichstrasse 35, A-6020 Innsbruck, Austria

Tel: +4351250480958; Fax: +435125046780951; Email: [milospetrik@seznam.cz](mailto:milospetrik@seznam.cz)

Clemens Decristoforo

Clinical Department of Nuclear Medicine, Anichstrasse 35, A-6020 Innsbruck, Austria

Tel: +4351250480951; Fax: +435125046780951; Email: [Clemens.Decristoforo@uki.at](mailto:Clemens.Decristoforo@uki.at)
